# Dissociation of thyrotropin receptor function and thyrotropin dependency in rat thyroid tumour cell lines derived from FRTL-5.

**DOI:** 10.1038/bjc.1996.409

**Published:** 1996-08

**Authors:** C. J. van der Kallen, J. H. Coes, J. P. van Grafhorst, E. M. Schuuring, F. A. Ossendorp, J. H. Thijssen, M. A. Blankenstein, T. W. de Bruin

**Affiliations:** Department of Endocrinology, Utrecht University, The Netherlands.

## Abstract

**Images:**


British Journal of Cancer (1996) 74, 606-612
© 1996 Stockton Press All rights reserved 0007-0920/96 $12.00

### Dissociation of thyrotropin receptor function and thyrotropin dependency in rat thyroid tumour cell lines derived from FRTL-5

CJH van der Kallen[1], JH Coes[1], JP van Grafhorst[1], EMD Schuuring[2], FA Ossendorp[2], JHH Thijssen[1], MA Blankenstein[1] and TWA de Bruin[1]

[1]*Department of Endocrinology, Utrecht University and Academic Hospital Utrecht, HP GO2.625, PO Box 85500, NL-3508 GA Utrecht, The Netherlands;* [2]*Academic Hospital Leiden, Leiden, The Netherlands.*

**Summary** Spontaneously transformed somatic thyrocyte mutants, FRTL-5/TA and FRTL-5/TP, are thyrotropin (TSH) independent for growth and show loss of the thyroid-specific phenotype, with absent thyroglobulin and thyroid peroxidase gene expression. To investigate the role of TSH-receptor (TSH-R) activation in rat thyroid growth and function, binding of TSH and TSH-induced cAMP production were measured in intact cells under identical assay conditions. TSH binding did not differ in terms of affinity and receptor number and presence of 5.6 kb and 3.3 kb mRNA rat TSH-R transcripts was determined in all variants. By contrast, basal cAMP was 11-fold lower in FRTL-5/TA and 6-fold lower in FRTL-5/TP than in wild-type FRTL-5 ($1.1 \pm 0.4$; $P<0.01$). Maximal cAMP production was similar between wild-type and cell variants and stimulation by bovine, rat and recombinant human TSH revealed normal activation patterns. Therefore, a dissociation was present between the loss of TSH control on growth and function, and the presence of a normally functioning TSH-R. Subsequent to TSH incubation FRTL-5/TP and FRTL-5/TA cells showed a different expression pattern of TSH-R and the proto oncogenes c-*myc* and *fos* than FRTL-5 wild-type. The data indicated that the cause of the TSH-independency is located down-stream of the cAMP cascade, influencing genes that control the expression of cell cycle-related proto-oncogenes and thyroid-specific genes.

**Keywords:** thyroid; thyrotropin; thyrotropin receptor; cAMP; FRTL-5

The glycoprotein hormone thyrotropin (TSH) is the major regulator of thyroid function and growth (Vassart and Dumont, 1992). Actions of TSH are exerted through interaction with the plasma membrane TSH receptor (TSH-R), which has been characterised as a member of the G-protein coupled receptor family (Nagayama *et al.*, 1989; Libert *et al.*, 1989). Following binding of TSH to the TSH-R, G-proteins will be activated, resulting in stimulation of adenylate cyclase (Dumont *et al.*, 1989) and cAMP production or in stimulation of phospholipase C with subsequent diacylglycerol and inositol-3-phosphate production (Vassart and Dumont, 1992). cAMP is generally assumed to regulate a major part of thyroid function and thyroid growth (Vassart and Dumont, 1992).

Thyroid tumours can be divided into functional and non-functional tumours. With regard to growth and metabolic activity, functional tumours are responsive to TSH like normal thyroid tissue. As a tumour evolves, phenotype-specific metabolic activities decrease or disappear (Christov and Raichev, 1972; Wollman, 1963), as has been described in transgenic models (Ledent *et al.*, 1991). Non-functional tumours show absence of phenotype-specific thyroid functions and, characteristically, TSH is unable to stimulate such functions, or growth. Therefore, loss of TSH responsiveness could result from a TSH-R defect or a defect in a post-receptor element important for signal transduction. For example, absence of high-affinity TSH-R (Abe *et al.*, 1981), a defective coupling between the TSH-R and G-protein (Namba *et al.*, 1993), a TSH-R point mutation (Parma *et al.*, 1993), and a $G_s\alpha$ mutation including those found in other endocrine tumours (Lyons *et al.*, 1990; Yoshimoto *et al.*, 1993) have been suggested to explain the observed TSH independency or TSH unresponsiveness. In experimental terms validity of either of the first two possibilities would be supported by findings of abnormal TSH binding or an abnormal pattern of TSH-induced cAMP production. The latter two possibilities are then expected to result in a higher basal cAMP level.

In the present study we tested these possibilities experimentally using the FRTL-5 rat thyroid cell line and spontaneously transformed somatic cell variants with different degrees of differentiation and sensitivity to TSH (Ossendorp *et al.*, 1990). FRTL-5 cells are an accepted model for normal thyroid function as these cells show responsiveness to TSH for iodide uptake, thyroglobulin (Tg) iodination and secretion, and cell growth. The variant cell lines have been generated by subcutaneous injection of FRTL-5 cells in nude mice. From the tumours that subsequently developed, variant cell lines were derived as described by Ossendorp *et al.* (1990). The variant cell line FRTL-5/T, isolated from a functionally active tumour, shows normal responsiveness to TSH for most thyroid-specific functions (Ossendorp *et al.*, 1990). This is in contrast to the FRTL-5/TA and FRTL-5/TP variants, isolated from non-functional tumours, which are insensitive to TSH with respect to iodide uptake, thyroglobulin iodination and secretion, and cell growth, thus fulfilling the criteria of somatic cell mutants with the phenotype of autonomous TSH-independent growth and function (Ossendorp *et al.*, 1990). In the present study we evaluated whether acquisition of autonomy was associated with defects in the binding of the ligand, or with defects in the second messenger system. The production of cAMP in response to TSH was characterised under the same conditions in which TSH binding to its receptor can be measured; binding was evaluated in terms of capacity and affinity. In addition, the response of the proto-oncogenes c-*myc* and *fos* was measured to characterise the effect of TSH at the early steps of the cell cycle.

### Materials and methods

### *Culture of FRTL-5 cells and variant cell lines*

FRTL-5, FRTL-5/T and FRTL-5/TP cells were cultured in 6H (six-hormone mixture) medium (Ambesi-Impiombato *et al.*, 1980) consisting of Coon's modified Ham's medium

Correspondence: CJH van der Kallen
Received 23 November 1995; revised 22 February 1996; accepted 8 March 1996

(Gibco BRL, Breda, The Netherlands) containing 5% fetal calf serum (Gibco) and insulin (10 $\mu$g ml$^{-1}$), hydrocortisone (0.36 ng ml$^{-1}$), transferrin (5 $\mu$g ml$^{-1}$), somatostatin (10 ng ml$^{-1}$), glycyl-L-histidyl-L-lysine acetate (2 ng ml$^{-1}$) (all Sigma, St Louis, MO, USA) and bTSH (bovine TSH) 100 $\mu$U ml$^{-1}$ (Ambinon, Organon, Oss, The Netherlands). As FRTL-5/TA cells were generated under TSH-free conditions, the cells were cultured in 5H (5 hormone mixture) medium (TSH-free medium, identical to the medium described above but without TSH) as described previously (Ossendorp *et al.*, 1990).

In experiments that measured cAMP production and TSH binding, cells were cultured in six-well plates (Gibco). Five days before the experiments, cell variants were cultured without TSH in such a way that the cells were confluent on the experimental day. One day before the experiments fresh medium was added to the cells.

### *[$^{125}$I]TSH binding and competition (whole cell assay)*

The iodination of bTSH (kindly supplied by Dr JG Pierce, University of California, CA, USA) was performed as described by Roelen *et al.* (1992) for human growth hormone. Briefly, 5 $\mu$g of bTSH was iodinated in a total volume of 25 $\mu$l with 0.3 mg ml$^{-1}$ chloramine-T (final concentration), the reaction was terminated with 100 $\mu$l of 0.96 mg ml$^{-1}$ Na-meta-bisulphite (final concentration). Free and bound $^{125}$I were separated on Sephadex G-25 (PD-10 columns, Pharmacia, Uppsala, Sweden). The incorporation of $^{125}$I was 72$\pm$6.4% (mean$\pm$s.e.m., $n$=9). The [$^{125}$I]bTSH in the protein fraction obtained from the Sephadex G-25 column was receptor purified by binding to and elution from porcine thyroid plasma membranes (Smith *et al.*, 1977). Eluates were purified on a Sephadex S-200 column with Tris sodium chloride (10–50 mM) containing 0.1% bovine serum albumin (BSA).

Confluent cells were washed three times with modified Hanks' balanced salt solution (HBSS: 5 mM potassium chloride, 1.3 mM calcium chloride, 0.4 mM magnesium sulphate, 0.34 mM disodium hydrogen phosphate, 0.44 mM potassium hydrogen phosphate, 280 mM sucrose and 0.25% BSA) and incubated for 1 h at 37°C in 1 ml of HBSS containing [$^{125}$I]bTSH ($\pm$10 000 c.p.m. sp. act. 1.2 $\times$ $10^6$ d.p.m. fmol$^{-1}$) and various concentrations of bTSH (1 $\mu$U = 2.4 fmol), rhTSH (recombinant human TSH, Genzyme, West-Malling, UK; 1 $\mu$U = 4.2 fmol) and rTSH (rat TSH, kindly provided by NIDDK, NHPP, Baltimore, MD, USA; 1 $\mu$U = 1.0 fmol) (0–2.40, 0–0.42 and 0–0.24 $\mu$M respectively). After 1 h the incubation buffer was removed, cells were washed three times with ice-cold HBSS and 1 ml of 1 N sodium hydroxide was added to detach the cells. The bound radioactivity was transferred to tubes and counted in a $\gamma$-counter. The dissociation constants ($K_d$) of bTSH and the number of TSH binding sites (R) were calculated with a linear subtraction method (Van Zoelen, 1989). To compare the affinities of bTSH, rTSH and rhTSH $IC_{50}$-values (50% competition) were determined from the competition curves.

### *Effect of TSH on intracellular cAMP response*

Confluent cells were washed three times with HBSS. The cells were incubated at 37°C in 1 ml of HBSS containing 0.5 mM 3-isobutyl-1-methylxanthine (Sigma) and various concentrations of bTSH, rhTSH, rTSH (0–0.24, 0–0.44 and 0–0.024 $\mu$M respectively). After 1 h the incubation was terminated by removing the buffer and addition of 1 ml of 96% ice-cold ethanol. Cells were stored at least overnight at −20°C. Cells were scraped with a rubber policeman, the ethanol was evaporated at 37°C under a stream of nitrogen and 0.5 ml of a Tris-EDTA (50 mM, 4 mM, pH 7.5) buffer was added. After centrifugation for 10 min at 3600 r.p.m. the supernatants were used to measure cAMP (radioreceptor assay TRK 432, Amersham, Little Chalfont, UK). DNA was measured in the pellets (Burton, 1956), using calf thymus DNA (Boehringer Mannheim, Mannheim, Germany) as a standard. To compare the effects of bTSH, rTSH and rhTSH, their concentrations yielding 50% of the maximal cAMP response ($EC_{50}$) were calculated from the dose–response curves.

Measurements of intracellular cAMP were performed under similar conditions that enabled assay of TSH binding and competition. Control experiments, comparing cells of several passages, revealed similar properties with regard to TSH binding and cAMP response in the course of 4 years with all variants and wild-type FRTL-5.

### *Detection of TSH-R,* c-myc *and fos RNA expression*

FRTL-5 cells and variants were harvested for RNA isolation after 7 days of TSH depletion (5H medium) or after variable intervals following TSH stimulation (100 $\mu$U ml$^{-1}$). RNA isolation was performed as described previously (Ossendorp *et al.*, 1990). In short, cells were homogenised in 6 M urea/3 M lithium chloride. After centrifugation (10 000 $\times$ *g*, 45 min, 4°C), the pellet was dissolved in 10 mM Tris-HCl (pH 7.6), 5 mM EDTA, 1% sodium dodecyl sulphate (SDS), deproteinised by two extracts with phenol-chloroform (1:1) and precipitated with ethanol. Samples of RNA were denatured at 60°C for 30 min in a solution containing 50% (v/v) formamide, 2.2 M formaldehyde and RNA running buffer (20 mM) MOPS (pH 7.0), 5 mM sodium acetate, and 1 mM EDTA.

For Northern analysis, RNA samples were separated by electrophoresis in 1.0% agarose gels, followed by transfer to nitrocellulose filters and fixed by heating (80°C for 4 h) as described in detail (Ossendorp *et al.*, 1990). Membranes were hybridised with $^{32}$P-labelled probes (Ossendorp *et al.*, 1990). The following probes were used: cDNA clones of the c-*myc* and *fos* genes (Tramontano *et al.*, 1986) and a prepared cDNA rat TSH-R insert (1039–1190) probe. The TSH-R cDNA probe (1039–1190) was prepared by polymerase chain reaction (PCR) of the T8AFB clone (courteously donated by Dr Leonard Kohn, NIH, Bethesda, MD, USA) containing the full-length rat cDNA coding sequence (−54 to 2780 bp). For reprobing the filters were stripped by washing the filter twice with 0.05 $\times$ SSC, 0.01 M EDTA (hot) and 0.1% SDS for 15 min and once briefly with 0.01 $\times$ SSC at room temperature.

Ethidium bromide staining of whole RNA in the agarose gel revealed the 28S (4.8 kb) and 18S (2.0 kb) ribosomal RNA markers respectively, which were used as a control for application of equal amounts of RNA. Quantification of intensity was performed by densitometer.

### *Statistical analysis*

Results are given as means$\pm$s.e.m. ($n$). Significances of the observed differences were calculated with the non-parametric Mann–Whitney $U$-test. $P$-values $\leqslant$0.05 were considered to reflect statistical significance.

### Results

### *[$^{125}$I]TSH binding and competition (whole cell assay) (Figure 1 and 2, Table I)*

Total binding of [$^{125}$I]bTSH to FRTL-5 cells was 8.3$\pm$1.4% of the amount added ($n$=8), with a non-specific binding of 0.31$\pm$0.12%. These values did not differ significantly between the different cell line variants. The combined data of the competition curves and Scatchard plot analysis of FRTL-5 cells ($n$=7–8, separate experiments) are shown in Figures 1a and 2. The analysis was performed both for one and two classes of binding sites. Analysis according to a two-class model yielded a high-affinity, low-capacity binding site with properties similar to the results of the analysis for one binding site. In addition, a binding site with extremely low affinity and high capacity was detectable. As this binding site was considered to represent non-specific binding, further

**Figure 1** cAMP response to bTSH ($n=5-7$) and [$^{125}$I]bTSH competition curve ($n=7-8$) of FRTL-5 (**a**), FRTL-5/T (**b**), FRTL-5/TP (**c**) and FRTL-5/TA cells (**d**). Cells are incubated for 1 h (HBSS) with different concentrations of bTSH. B/Bo (%) and cAMP (%) represent the values measured relative to the maximal values; ●, TSH displacement; +, cAMP response.

**Table I** Capacity (R) and affinity ($K_d$) of bTSH binding to FRTL-5 wild type and variant cell lines and $IC_{50}$-values for the competition of [$^{125}$I]bTSH by radioinert competitors

| | *Cell line* | | | |
|---|---|---|---|---|
| *Parameter* | *FRTL-5* | *FRTL-5/T* | *FRTL-5/TP* | *FRTL-5/TA* |
| (R) | 134±20 (8) | 259±93 (5) | 127±28 (5) | 102±29 (7) |
| $K_d$ bTSH | 1.0±0.4 (8) | 2.4±0.5 (5) | 1.8±0.3 (5) | 2.3±0.5 (8) |
| $IC_{50}$ bTSH | 2.7±0.6[a] (8) | 2.7±0.7[a] (5) | 3.6±1.1[a] (5) | 3.7±0.7[a] (7) |
| $IC_{50}$ rTSH | 2.0±0.9[a] (6) | 4.3±1.4 (4) | 1.9±1.3[a] (3) | 1.9±0.5[a] (4) |
| $IC_{50}$ rhTSH | 56±24 (3) | 35±19 (3) | 127±22[b] (3) | 103±34 (3) |

$K_d$ and $IC_{50}$ are given in nM, (R) in fmol/well. $K_d$ and R are calculated based on the presence of one binding site. Values represent means±s.e.m. ($n$). [a]Significantly different ($P<0.05$) *vs* rhTSH. [b]Significantly different ($P<0.05$) *vs* FRTL-5/T.

analysis is only reported for the one class model (Table I). Neither the $K_d$ (1.0–2.4 nM) nor the receptor number (R) differed significantly among the different cell lines. Competition by TSH from several species (rat, bovine and recombinant human) showed a similar pattern between cell variants (Figure 1). Differences between TSH species were noted, i.e. the $IC_{50}$ value of rhTSH was approximately one order of magnitude higher than the $IC_{50}$ value of bTSH and rTSH, by contrast the $IC_{50}$ value of rTSH did not differ from $IC_{50}$ found with bTSH (Table I).

*Measurement of cAMP response to TSH (Figure 1, Table II)*

The basal intracellular cAMP level was 11-fold lower in FRTL-5/TA cells and 5.8-fold lower in FRTL-5/TP cells than in wild-type FRTL-5 (1.1±0.35 pmol cAMP $\mu g^{-1}$ DNA). In contrast a cAMP response was elicited by TSH to the same maximal level in each cell variant (7.9–11.1 pmol cAMP $\mu g^{-1}$ DNA) by 240 nM. Thus, the induction of cAMP production by TSH is different between wild-type (10-fold induction) and the TSH-independent growing cell lines FRTL-5/TP and FRTL-5/TA (64- to 79-fold induction). Similar $EC_{50}$ values were obtained with rTSH and bTSH. The $EC_{50}$ value of rhTSH was approximately one order of magnitude higher than the $EC_{50}$ value of bTSH and rTSH. The significant differences between rhTSH $IC_{50}$ and $EC_{50}$ values, observed in the variant cell lines, was probably caused by the use of two different batches of rhTSH. Different batches of rhTSH have different degrees of sialylation and sulphation, causing different TSH bioactivity (Szkudlinski *et al.*, 1993).

*Northern blotting (Figure 3)*

The rat TSH-R-specific probe hybridised with a 5.6 and 3.3 kb mRNA signal respectively, following Northern blotting. These sizes of mRNA transcripts have been identified in FRTL-5 cells as transcripts of the rat TSH-R (Akamizu *et al.*, 1990). In all variants normal sized TSH-R mRNA transcripts were observed (Figure 3), although the intensity differed. This was confirmed by densitometry. Culture of FRTL-5 cells with TSH (6H) down-regulated TSH-R mRNA and this effect was also demonstrated in

**Figure 2** Scatchard analysis of the competition curve (Figure 1a) of FRTL-5 cells ($n = 7-8$).

**Figure 3** Northern blot analysis of TSH-R, *fos* and c-*myc* mRNA in FRTL-5 and variant cells and effect of the presence of TSH (100 $\mu$U ml$^{-1}$). 5H, culture without TSH; 6H, culture in the continuous presence of TSH; 40 min and 24 h reflect times following addition of TSH. The experimental protocol used is described in detail in the Materials and methods.

TSH-dependent FRTL-5/T cells. In FRTL-5/TP and FRTL-5/TA (TSH-independent variants) the intensity of the TSH-R transcript was substantially lower in the absence of TSH (5H) and stimulation by TSH did not lead to a decreased intensity.

Addition of TSH to culture medium resulted in the increased expression of c-*myc* and *fos* mRNA transcripts, although the time course and the c-*myc*/*fos* mRNA signal ratio in FRTL-5/TP and FRTL-5/TA differed from that observed in FRTL-5 wild-type. In FRTL-5 cells both *fos* and c-*myc* mRNA expression was transiently increased after 40 min of stimulation by TSH, c-*myc* was still elevated after 24 h. Although the 40 min point is missing, the FRTL-5/T variant showed the same expression pattern as the FRTL-5/T cells; *myc* was elevated at 24 h. In contrast, *myc* mRNA expression in FRTL-5/TP and FRTL-5/TA variants was lower and only slightly influenced by TSH. The expression of *fos* was stimulated in the FRTL-5/TP variant after 24 h stimulation by TSH, in FRTL-5/TA cells after 40 min stimulation by TSH.

### Discussion

In the present study we characterised functional TSH binding, cAMP response, TSH-R gene expression, and c-*myc*/*fos* proto-oncogene response in rat thyrocytes. As a model we used stable rat FRTL-5/T, FRTL-5/TP and FRTL-5/TA thyroid cell lines that spontaneously originated from wild-type FRTL-5 cells transplanted into nude mice and that were characterised by TSH-independent growth in combination with dedifferentiation (Ossendorp *et al.*, 1990). The functional binding of radiolabelled TSH, characterised by $K_d$ and number of binding sites, as well as $IC_{50}$ values of rat TSH and bovine TSH, were similar between TSH-dependent (FRTL-5 and T cells) and TSH-independent (TP and TA) cells. Because of the combination of low-affinity ($K_d$ 101.5 nM) and an exceptionally high number of binding sites per cells ($\pm 1.1 \times 10^6$), we concluded that this class of low-affinity binding sites represents non-specific binding. This is in agreement with other reports (Chazenbalk *et al.*, 1990; Nagayama and Rapoport, 1992), which concluded that this class of binding sites is an artefact because both untransfected and TSH-R transfected CHO cells show the same low-affinity site. The FRTL-5/TP and FRTL-5/TA variants showed normal maximal cAMP response, but 6- to 11-fold lower basal intracellular cAMP concentrations than wild-type FRTL-5. The $EC_{50}$ values obtained with rat and bovine TSH were similar between all variants and wild-type FRTL-5. Assays of TSH binding and TSH-induced cAMP production were carried out under identical conditions of cell culture, excluding artefacts which might be caused by low-salt and high-salt culture conditions (Tramontano and Ingbar, 1986). The present findings therefore formally demonstrate the dissociation between presence of a functionally intact TSH-R, able to activate the cAMP cascade and TSH control on the growth and differentiation in FRTL-5/TP and FRTL-5/TA cells.

The exact role of TSH in the control of thyroid growth is still unresolved because thyroid mitogenic and anti-mitogenic effects have been reported in different species (Maenhaut *et al.*, 1990; Vassart and Dumont, 1992). TSH is a proven growth factor in FRTL-5 cells (Jin *et al.*, 1986; Coletta *et al.*, 1986), dog thyrocytes (Maenhaut *et al.*, 1990) and human thyrocytes (Dumont *et al.*, 1992). TSH is able to activate

**Table II** cAMP response to bTSH in FRTL-5 wild type and sublines and $EC_{50}$ values of different species of TSH

| | *Cell line* | | | |
|---|---|---|---|---|
| *Parameter* | *FRTL-5* | *FRTL-5/T* | *FRTL-5/TP* | *FRTL-5/TA* |
| Basal cAMP | $1.1 \pm 0.4$ (7) | $0.38 \pm 0.18$[a] (5) | $0.19 \pm 0.08$[b] (5) | $0.10 \pm 0.03$[b] (6) |
| Max cAMP | $11.1 \pm 2.0$ (7) | $11.5 \pm 3.8$ (5) | $12.2 \pm 3.4$ (5) | $7.9 \pm 2.3$ (6) |
| $EC_{50}$ bTSH | $0.22 \pm 0.05$[c] (7) | $0.22 \pm 0.05$[c] (7) | $0.26 \pm 0.08$[c] (5) | $0.29 \pm 0.08$[c] (6) |
| $EC_{50}$ rTSH | $0.28 \pm 0.11$[c] (4) | $0.44 \pm 0.09$[c,d] (4) | $0.90 \pm 0.80$[c] (3) | $0.44 \pm 0.16$[c] (3) |
| $EC_{50}$ rhTSH | $2.1 \pm 0.6$[e] (3) | $10.1 \pm 5.9$ (3) | $5.8 \pm 1.2$[f] (4) | $14.3 \pm 1.8$ (3) |

Max cAMP reflects cAMP production at TSH 240 nM, cAMP values are given in pmol $\mu$g$^{-1}$ DNA, $EC_{50}$ values are given in nM, values represent means ± s.e.m. ($n$). [a]Significantly different ($P < 0.05$) *vs* FRTL-5/TA. [b]Significantly different ($P < 0.01$) *vs* FRTL-5. [c]Significantly different ($P < 0.05$) *vs* rhTSH. [d]Significantly different ($P < 0.05$) *vs* bTSH. [e]Significantly different ($P < 0.05$) with the other cell variants. [f]Significantly different ($P < 0.05$) *vs* FRTL-5/TA.

both the cAMP cascade and phospholipase C pathway independently by binding to its receptor (Maenhaut *et al.*, 1990; Fujimoto and Brenner-Gatti, 1992). The current theory is that enhancement of the cAMP cascade relates to stimulation and control of functional characteristics in human and FRTL-5 (rat) thyrocytes, including iodide uptake and expression of thyroid-specific proteins thyroglobulin and thyroid peroxidase (Maenhaut *et al.*, 1990). In agreement with this theory is the recent demonstration that gain-of-function mutations in the TSH-R cause hyperfunctioning thyroid adenomas (Parma *et al.*, 1993) and congenital hyperthyroidism (Kopp *et al.*, 1995), which are characterised by higher basal intracellular cAMP concentrations in agreement with the original report by Kasagi *et al.* (1980) in a collection of human adenoma tissues. Dedifferentiating tumours may then be caused by activation of growth factor and phorbol ester cascades (Maenhaut *et al.*, 1990; Mockel *et al.*, 1994), and not through activation of cAMP cascade. The finding of reduced basal cAMP concentrations in dedifferentiated FRTL-5/TP and FRTL-5/TA cells, a factor 6 to 11 lower than FRTL-5 cells, therefore represents novel evidence to support this theory, and possibly indicates a mechanism of inverse agonism, causing a reduction in the basal regulation of the adenylate cyclase (Milligan *et al.*, 1995). FRTL-5/TA and FRTL-5/TP cells have lost most of the thyroid-specific phenotype of wild-type FRTL-5 cells as demonstrated by virtual absence of iodide uptake, and absence of thyroglobulin and TPO mRNA (Ossendorp *et al.*, 1990). In addition, FRTL-5/TP and FRTL-5/TA cells demonstrate a dissociation between intact TSH-R function in the cAMP cascade and loss of TSH control over thyroid growth. Thus, acquisition of dominant signal transduction pathways other than the cAMP cascade, or in theory further downstream in the cAMP cascade, is relevant and advantageous for thyrocytes to develop autonomous growth and dedifferentiation.

The present findings do not support the claim by Berlingeri *et al.* (1990) that the loss of TSH control on growth correlates with the complete loss of rat TSH-R gene expression. Using the same rat TSH receptor cDNA probe, these authors could not detect TSH-R mRNA in oncogene transformed FRTL-5 cells; functional TSH binding was not measured. We detected TSH-R mRNA in TSH-independent FRTL-5/TP and FRTL-5/TA variants, although the gene expression was lower than the corresponding transcript signal in FRTL-5 cells. Interestingly, this reduction in TSH-R gene expression did not affect the binding characteristics of radiolabelled TSH. The present data indicated that TSH-R mRNA does not correspond to the number of functioning receptors, suggesting: (1) that degradation or stability of mRNA species (5.6 and 3.3 kb) is different between cell variants; (2) TSH-R protein may have a larger residence time on the cell surface; and (3) a functional assay of TSH binding needs to be combined with measurement mRNA TSH-R levels. In the literature, species-specific responses of TSH-R mRNA expression to TSH exposure have been reported: down-regulation in rat thyrocytes (Akamizu *et al.*, 1990; and the present findings), a slight down-regulation in dog thyrocytes and no marked down-regulation in human thyrocytes (Maenhaut *et al.*, 1992). Significantly reduced TSH-R gene expression has also been found in neoplastic human thyroid tissues in conjunction with reduced thyroglobulin gene expression (Ohta *et al.*, 1990; Elisei *et al.*, 1994). However, several laboratories have shown that functional TSH binding is, in general, not different between normal and neoplastic human thyroid tissues (Karlsson and Dahlberg, 1979; Matsuo *et al.*, 1993). Therefore, a similar discrepancy as found in FRTL-5/TP and FRTL-5/TA cells between reduced TSH-R gene expression and intact TSH binding can exist in human thyroid neoplasms.

Coletta *et al.* (1986) showed that TSH increased *fos* and c-*myc* gene expression in FRTL-5 cells under controlled conditions of thyrocyte growth. Our findings on *fos* and c-*myc* gene expression in FRTL-5 cells are similar to those by Coletta *et al.* (1986). and to the findings in dog thyrocytes (Reuse *et al.*, 1990); *fos* and c-*myc* gene expression were transiently stimulated after the addition of exogenous TSH to TSH-deprived FRTL-5 cells or T variant cells. Interestingly, in TSH-independent FRTL-5/TP and FRTL-5/TA variants addition of exogenous TSH was again associated with an increase in *fos* and c-*myc* gene expression, although the latter transcript signal was reduced compared with wild-type FRTL-5. Our data unequivocally show that the TSH-induced gene expression of *fos* and c-*myc* can be dissociated from the phenomenon of TSH-dependent growth and are in agreement with and extend the conclusion by Heldin and Westermark (1988) and Wyllie *et al.* (1989). Other possible mechanisms, known to activate the thyroid-specific genes *Tg*, *TPO* and *TSH-R*, are Pax-8 and thyroid transcription factor 1 and 2 (Zannini *et al.*, 1992; Civitareale *et al.*, 1993; Shimura *et al.*, 1994). Interestingly, transformation of FRTL-5 cells with viral oncogenes (Ki-*ras*, Ha-*ras* and *mos*) leads to undetectable expression of *Tg* and *TPO* genes (as in the FRTL-5/TP and FRTL-5/TA variants), in combination with absence of Pax-8 expression (Francis-Lang *et al.*, 1992). Further studies are planned to characterise the dominant signal transduction pathways in FRTL-5/TP and FRTL-5/TA cells and their effect on genes known to be involved in thyroid growth and function. In conclusion, a functionally normal TSH-R was found in FRTL-5 cells and its TSH-independent variants. Mutations in the TSH-R or $G_s\alpha$ are therefore unlikely explanations for the TSH independence in FRTL-5/TP and FRTL-5/TA cells. In these cells the functionally intact TSH-R is dissociated from the TSH control on growth and differentiation. Supported by the reduced basal levels of cAMP in FRTL-5/TP (6-fold) and FRTL-5/TA (11-fold), we conclude that a different mechanism, down-stream of the cAMP cascade, is responsible for a different activation pattern of *fos*, c-*myc* and *TSH-R* mRNA and the autonomous growth of FRTL-5/TP and FRTL-5/TA cells.

**Acknowledgements**
We thank Dr EJJ van Zoelen, who provided expert help with fitting procedures and courteously donated the mathematical program. Financial support was obtained from the PhD Research Fund of the Medical Faculty Utrecht University and the Jan Dekker and Dr Ludgardine Bouwman Fund.

### References

ABE Y, ICHIKAWA Y, MURAKI T, ITO K AND HOMMA M. (1981). Thyrotropin (TSH) receptor and adenylate cyclase activity in human thyroid tumors: absence of high affinity receptor and loss of TSH responsiveness in undifferentiated thyroid carcinoma. *J. Clin. Endocrinol. Metab.*, **52,** 23–28.

AKAMIZU T, IKUYAMA S, SAJI M, KOSUGI S, KOZAK C, MCBRIDE OW AND KOHN LD. (1990). Cloning of the rat thyrotropin receptor and thyrotropin-induced down regulation of the receptor in FRTL-5 cells. *Proc. Natl Acad. Sci. USA*, **87,** 5677–5682.

AMBESI-IMPIOMBATO FS, PARKS LAM AND COON HG. (1980). Culture of hormone-dependent functional epithelial cells from rat thyroids. *Proc. Natl Acad. Sci. USA*, **77,** 3455–3459.

BERLINGIERI MT, AKAMIZU T, FUSCO A, GRIECO M, COLLETTA G, CIRAIFICI AM, IKUYAMA S, KOHN LD AND VECCHIO G. (1990). Thyrotropin receptor gene expression in oncogene-transfected rat thyroid cells: correlation between transformation, loss of thyrotropin-dependent growth, and loss of thyrotropin gene expression. *Biochem. Biophys. Res. Commun.*, **173,** 172–178.

BURTON K. (1956). A study of the conditions and mechanism of the diphenylamine reaction for the colorimetric estimation of deoxyribonucleic acid. *Biochem.*, **62,** 315–323.

CHAZENBALK GD, NAGAYAMA Y, KAUFMAN KD AND RAPOPORT B. (1990). The functional expression of recombinant human thyrotropin receptors in nonthyroidal eukaryotic cells provides evidence that homologous desensitization to thyrotropin stimulation requires a cell-specific factor. *Endocrinology*, **127**, 1240–1244.

CHRISTOV K AND RAICHEV R. (1972). Experimental thyroid carcinogenesis. *Curr. Top. Pathol.*, **56**, 79–114.

CIVITAREALE D, CASTELLI MP, FALASCA P AND SAIARDI A. (1993). Thyroid transcription factor 1 activiates the promoter of the thyrotropin receptor gene. *Mol. Endocrinol.*, **7**, 1589–1595.

COLLETTA G, CIRAFICI AM AND VECCHIO G. (1986). Induction of the c-*fos* oncogene by thyrotropic hormone in rat thyroid cells in culture. *Science*, **233**, 458–460.

DUMONT JE, JAUNIAUX JC AND ROGER PP. (1989). The cyclic AMP-mediated stimulation of cell proliferation. *Trends Biochem. Sci.*, **14**, 67–71.

DUMONT JE, LAMY F, ROGER P AND MAENHAUT C. (1992). Physiological and pathological regulation of thyroid cell proliferation and differentiation by thyrotropin and other growth factors. *Phys. Rev.*, **72**, 667–691.

ELISEI R, PINCHERA A, ROMEI C, GRYCZYNSKA M, POHL V, MAENHAUT C, FUGAZZOLA L AND PACINI F. (1994). Expression of thyrotropin receptor (TSH-R), thyroglobulin, TPO and calcitonin messenger ribonucleic acids in thyroid carcinomas: evidence of TSH-R gene transcript in medullary histotype. *J. Clin. Endocrinol. Metab.*, **78**, 867–871.

FRANCIS-LANG H, ZANNINI M, DE FELICE M, BERLINIGIERI MT, FUSCO A AND DI LAURO R. (1992). Multiple mechanisms of interference between transformation and differentiation in thyroid cells. *Mol. Cell. Biol.*, **12**, 5793–5800.

FUJIMOTO J AND BRENNER-GATTI L. (1992). Protein kinase-C activation during thyrotropin-stimulated proliferation of rat FRTL-5 thyroid cells. *Endocrinology*, **130**, 1587–1592.

HELDIN NE AND WESTERMARK B. (1988). Epidermal growth factor, but not thyrotropin, stimulates expression of c-fos and c-myc messenger ribonucleic acid in porcine thyroid follicle cells in primary culture. *Endocrinology*, **122**, 1042–1046.

JIN S, HORNECK FJ, NEYLAN D, ZAKARIJA M AND MCKENZIE JM. (1986). Evidence that adenosine 3′, 5′-monophosphate mediates stimulation of thyroid growth in FRTL cells. *Endocrinol.*, **119**, 802–810.

KARLSSON FA AND DAHLBERG PA. (1979). Human thyrotropin receptors are expressed independently of the state of throid hormone production in thyroid tissue. *Horm. Metab. Res.*, **11**, 399–403.

KASAGI K, KONISHI J, ENDO K, MORI T, NAGAHARA K, MAKIMOTO K, KUMA K AND TORIZUKA K. (1980). Adenylate cyclase activity in thryoid tissue from patients with untreated Graves' disease. *J. Clin. Endocrinol. Metab.*, **51**, 492–499.

KOPP P, VAN SANDE J, PARMA J, DUPREZ L, GERBER H, JOSS E, JAMESON JL, DUMONT JE AND VASSART G. (1995). Congenital hyperthyroidism caused by a mutation in the thyrotropin receptor gene. *N. Engl. J. Med.*, **332**, 150–154.

LAURENT E, VAN SANDE J, LUDGATE M, CORVILAIN B, ROCMANS P, DUMONT JE AND MOCKEL J. (1991). Unlike thyrotropin, thyroid-stimulating antibodies do not activate phospholipase C in human thyroid cells. *J. Clin. Invest.*, **87**, 1634–1642.

LEDENT C, DUMONT J, VASSART G AND PARMENTER M. (1991). Thyroid adenocarcinomas secondary to tissue-specific expression of simian virus-40 large T-antigen in transgenic mice. *Endocrinology*, **129**, 1391–1401.

LIBERT F, LEFORT A, GERARD C, PARMENTER M, PERRET J, LUDGATE M, DUMONT JE AND VASSART G. (1989). Cloning, sequencing and expression of the human thyrotropin receptor: evidence for binding of autoantibodies. *Biochem. Biophys. Res. Commun.*, **165**, 1250–1255.

LYONS J, LANDIS CA, HARSH G, VALLAR L, GRÜNEWALD K, FIECHTINGER H, DUH Q-Y, CLARK OH, KAWASAKI E, BOURNE HR AND MCCORMICK F. (1990). Two G-protein oncogenes in human endocrine tumors. *Science*, **249**, 635–639.

MAENHAUT C, LEFORT A, LIBERT F, PARMENTIER M, RASPÉ E, ROGER B, LAURENT E, REUSE S, MOCKEL J, LAMY F, VAN SANDE J AND DUMONT JE. (1990). Function, proliferation and differentiation of the dog and human thyrocyte. *Horm. Metab. Res. Suppl.*, **23**, 51–61.

MAENHAUT C, BRABANT G, VASSART G AND DUMONT JE. (1992). *In vitro* and *in vivo* regulation of thyroprotein receptor mRNA levels in dog and human thyroid cells. *J. Biol. Chem.*, **267**, 3000–3007.

MATSUO K, FRIEDMAN E, GEJMAN PV AND FAGIN J. (1993). The thyrotropin receptor (TSH-R) is not an oncogene for thyroid tumors: structural studies of the TSH-R and the α-subunit of $G_s$ in human thyroid neoplasms. *J. Clin. Endocrinol. Metab.*, **76**, 1446–1451.

MILLIGAN G, BOND RA AND LEE M. (1995). Inverse agonism: pharmacological curiosity or potential therapeutic strategy. *Trends Pharmacol. Sci.*, **16**, 10–13.

MOCKEL J, LEJEUNE C AND DUMONT JE. (1994). Relative contribution to phosphoinositides and phophatidylcholine hydrolysis to the actions of carbamylcholine, thyrotropin, and phorbol esters on dog thyroid slices: regulation of cytidine monophosphate-phosphatidic acid accumulation and phospholipase-D activity. II. Actions of phorbol esters. *Endocrinology*, **135**, 2497–2503.

NAGAYAMA Y AND RAPOPORT B. (1992). The thyrotropin receptor 25 years after its discovery: new insight after its molecular cloning. *Mol. Endocrinol.*, **6**, 145–156.

NAGAYAMA Y, KAUFMAN KD, SETO P AND RAPOPORT B. (1988). Molecular cloning, sequencing and functional expression of the cDNA for the human thyrotropin receptors. *Biochem. Biophys. Res. Commun.*, **165**, 1184–1190.

NAMBA H, YAMASHITA S, USA T, KIMURA H, YOKOYAMA N, IZUMI M AND NAGATAKI S. (1993). Overexpression of the intact thyrotropin receptor in a human thyroid carcinoma cell line. *Endocrinology*, **132**, 839–845.

OHTA K, ENDO T AND ONAYA T. (1990). The mRNA levels of thyrotropin receptor, thyroglobulin and thyroid peroxidase in neoplastic human thyroid tissues. *Biochem. Biophys. Res. Commun.*, **174**, 1148–1153.

OSSENDORP FA, BRUNING PF, SCHUURING EMD, VAN DEN BRINK JAM, VAN DER HEIDE D, DE VIJLDER JJM AND DE BRUIN TWA. (1990). Thyrotropin dependent and independent thyroid cell lines selected from FRTL-5 derived tumors growth in nude mice. *Endocrinology*, **127**, 419–430.

PARMA J, DUPREZ L, VAN SANDE F, COCHAUX P, GERVY C, MOCKEL J, DUMONT J AND VASSART G. (1993). Somatic mutations in the thyrotropin receptor gene cause hyperfunctioning thyroid adenomas. *Nature*, **365**, 649–651.

REUSE S, MAENHAUT C AND DUMONT JE. (1990). Regulation of protooncogenes c-*fos* and c-*myc* expressions by protein tyrosine kinase, protein kinase C, and cyclic AMP mitogenic pathways in dog primary thyrocytes: a positive and negative control by cyclic AMP on c-myc expression. *Exp. Cell. Res.*, **189**, 33–40.

ROELEN CAM, DONKER GH, THIJSSEN JHH AND BLANKENSTEIN MA. (1992). A method for measuring the binding affinity and capacity of growth hormone binding protein in human serum using FPLC to separate bound and free ligand. *J. Liq. Chromatogr.*, **15**, 1259–1275.

SHIMURA H, OKAJIMA F, IKUYAMA S, SHIMURA Y, KIMURA S, SAJI M AND KOHN LD. (1994). Thyroid-specific expression and cyclic adenosine 3′, 5′-monophosphate autoregulation of the thyrotropin receptor gene involves thyroid transcription factor-1. *Mol. Endocrinol.*, **8**, 1049–1069.

SMITH BR, PYLE GA, PETERSEN VB AND HALL R. (1977). Interaction of thyrotropin with the human thyrotropin receptor. *J. Endocrinology*, **75**, 391–400.

SZKUDLINSKI MW, THOTAKURA NR, BUCCI I, JOSHI LR, TSAI A, EAST-PALMER J, SHILOACH J AND WEINTRAUB BD. (1993). Purification and characterization of recombinant human thyrotropin (TSH) isoforms produced by chinese hamster ovary cells: the role of sialylation and sulfation in TSH bioactivity. *Endocrinology*, **133**, 1490–1503.

TRAMONTANO D AND INGBAR SH. (1986). Properties and regulation of the thyrotropin receptor in FRTL-5 rat thyroid cell line. *Endocrinology*, **118**, 1945–1951.

TRAMONTANO D, CHIN WW, MOSES AC AND INGBAR SH. (1986). Thyrotropin and dibutyryl cyclic AMP increase levels of c-myc and c-fos mRNAs in cultured rat thyroid cells. *J. Biol. Chem.*, **261**, 3919–3922.

VAN ZOELEN EJJ. (1989). A new method for determining binding parameters without a priori assumptions on non-specific binding. *Biochem. J.*, **262**, 549–556.

VASSART G AND DUMONT JE. (1992). The thyrotropin receptor and the regulation to thyrocyte function and growth. *Endocrin. Rev.*, **13**, 596–611.

WESTERMARK B, KARLSSON FA AND WÄLINDER O. (1979). Thyrotropin is not a growth factor for human thyroid cells in culture. *Proc. Natl Acad. Sci. USA*, **76**, 2022–2026.

WOLLMAN SH. (1993). Production and properties of transplantable tumors of the thyroid gland in the Fischer rat. *Recent Prog. Horm. Res.*, **19**, 579–618.

WYLLIE FS, LEMOINE NR, WILLIAMS ED AND WYNFORD-THOMAS D. (1989). Structure and expression of nuclear oncogenes in multi-stage thyroid tumorigenesis. *Br. J. Cancer*, **60**, 561–565.

YOSHIMOTO K, IWAHANA H, FUKUDA A AND SANO T. (1993). Rare mutations of the Gs alpha subunit gene in human endocrine tumors. *Cancer*, **72**, 1386–1393.

ZANNINI M, FRANCIS-LANG H, PLACHOV D AND DI LAURO R. (1992). Pax-8, a paired domain-containing protein, binds to a sequence overlapping the recognition site of a homeodomain and activates transcription from two thyroid-specific promoters. *Mol. Cell. Biol.*, **12**, 4230–4241.


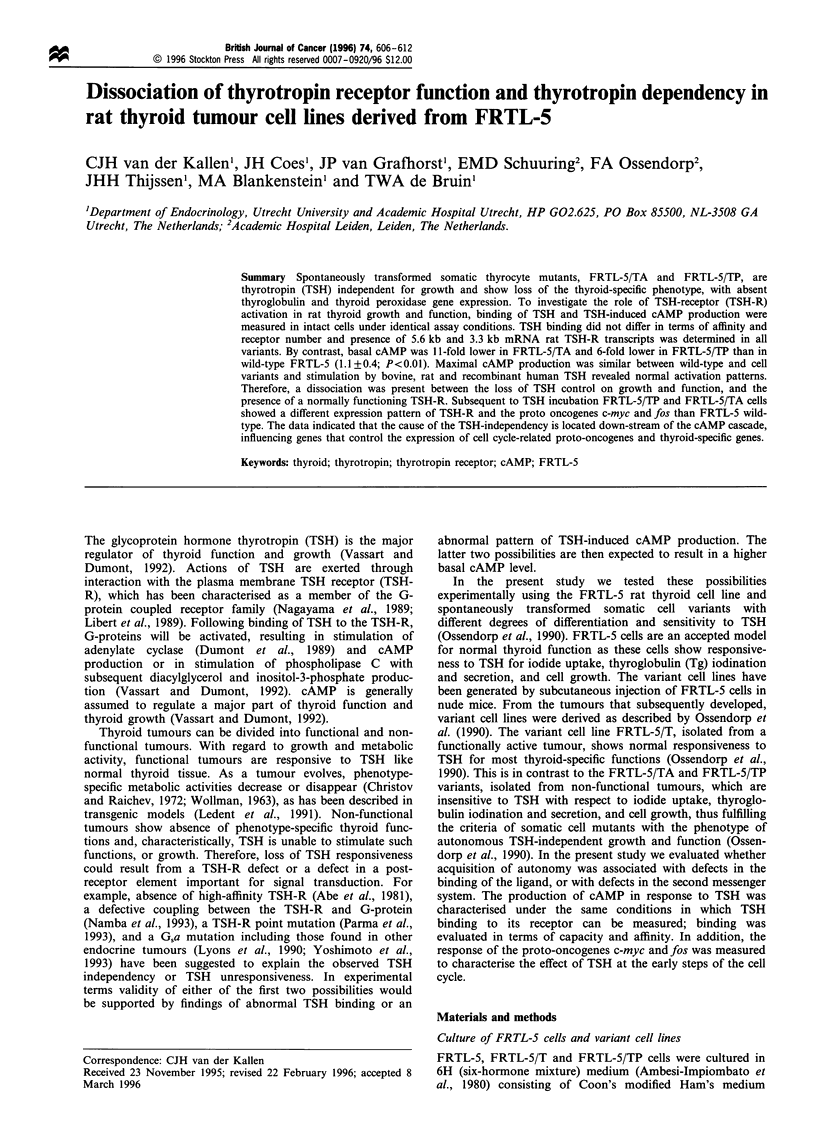

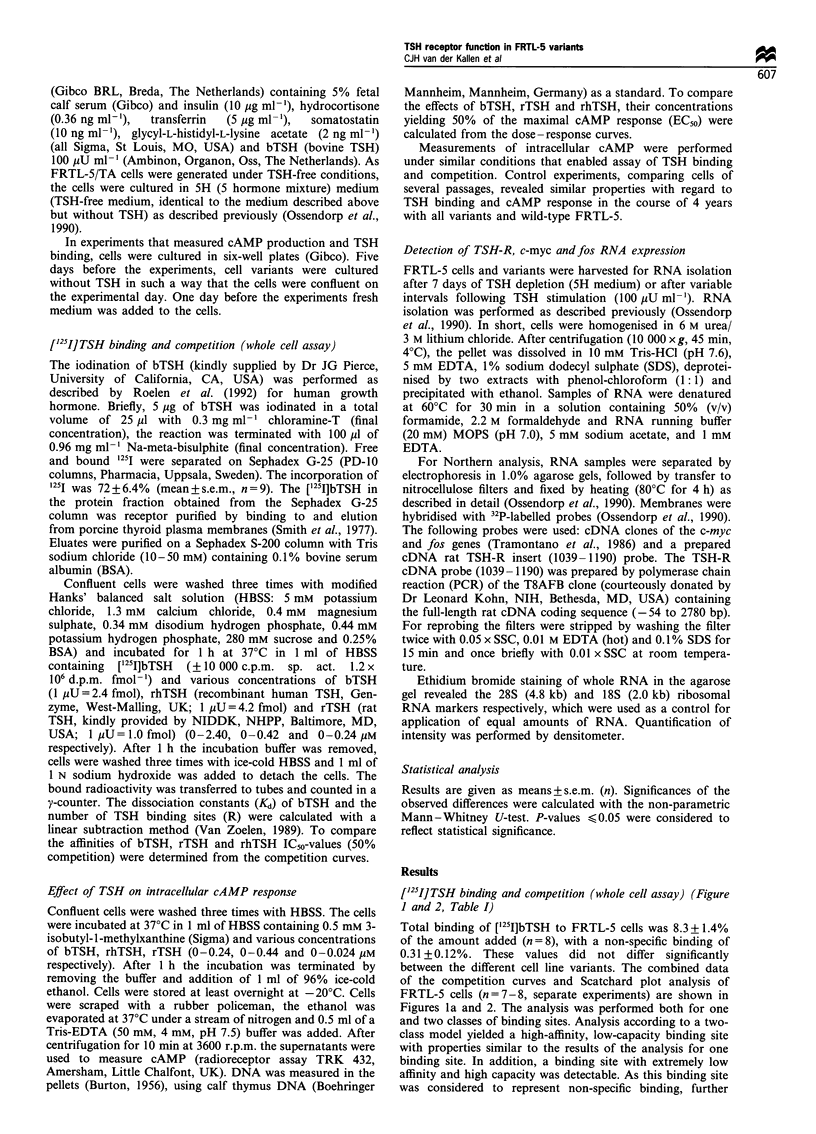

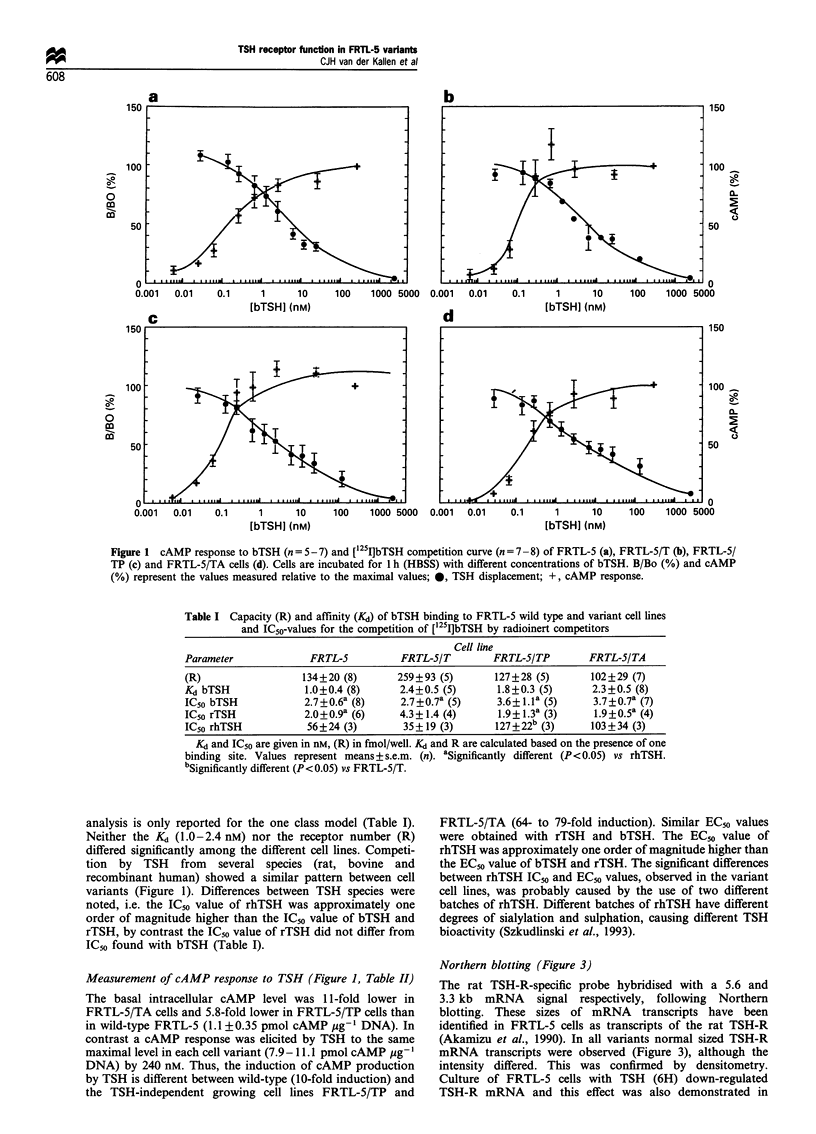

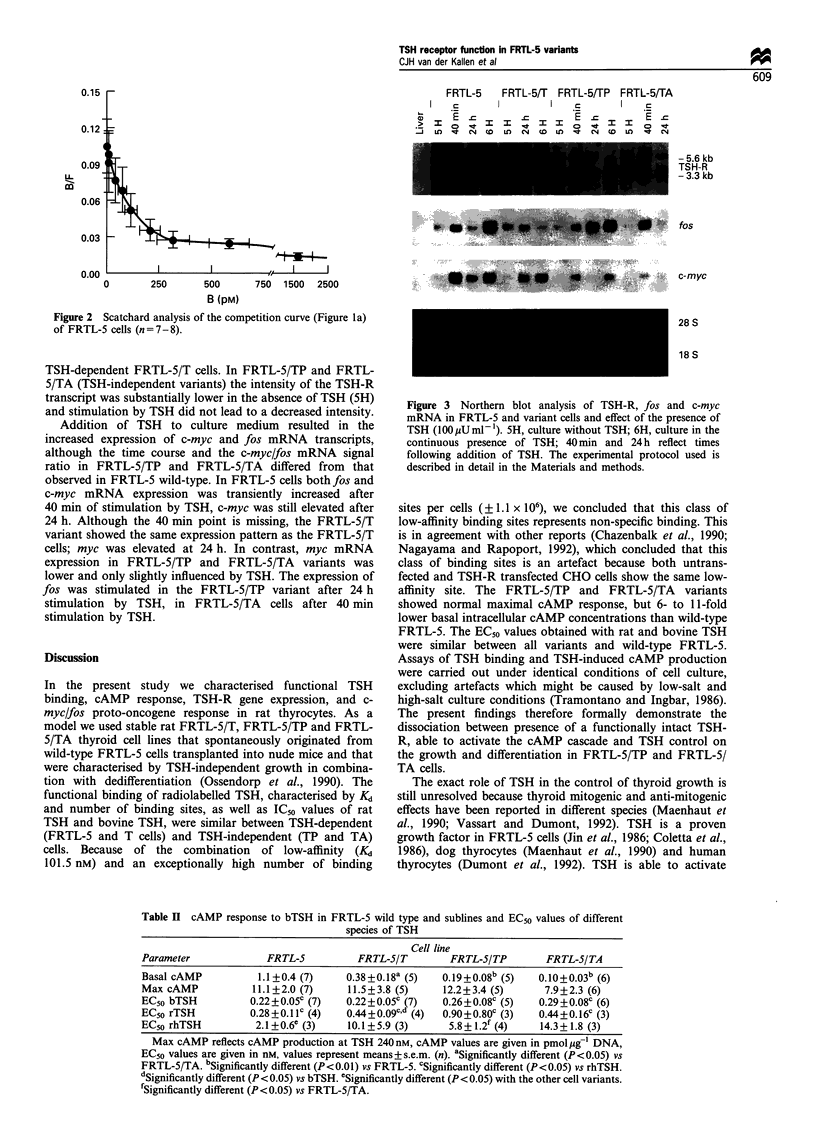

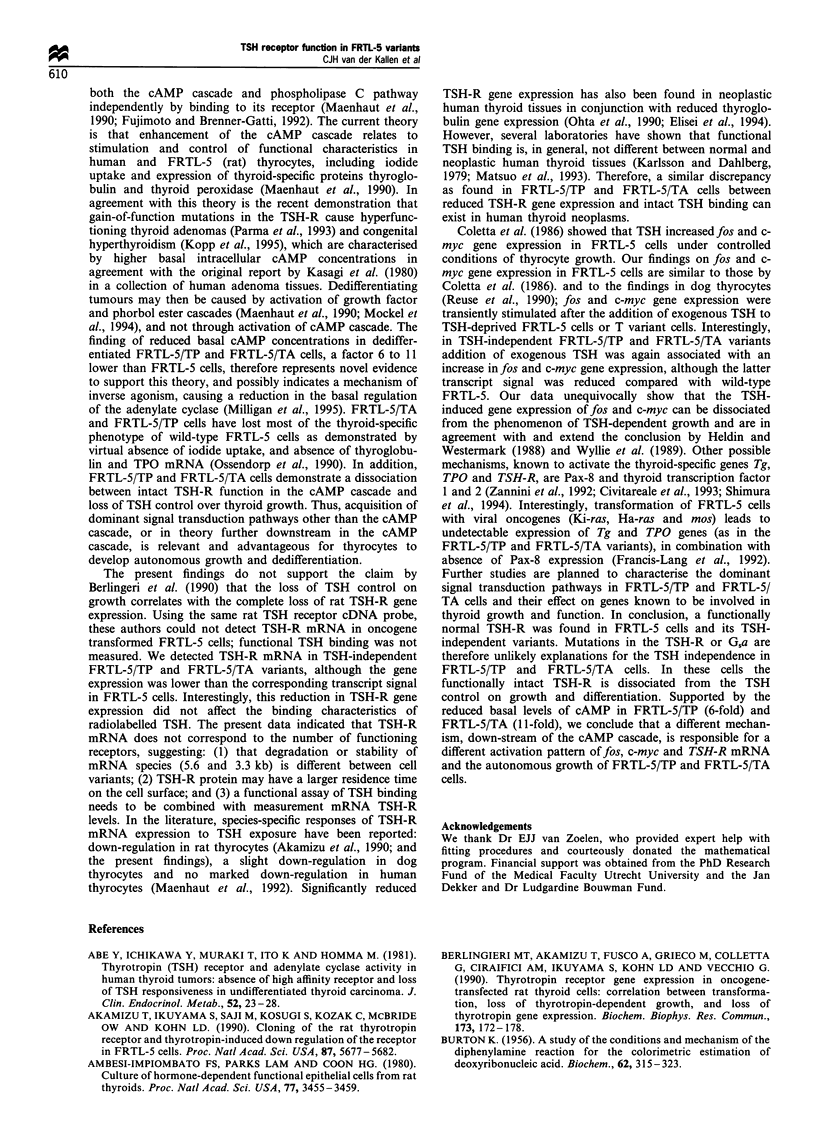

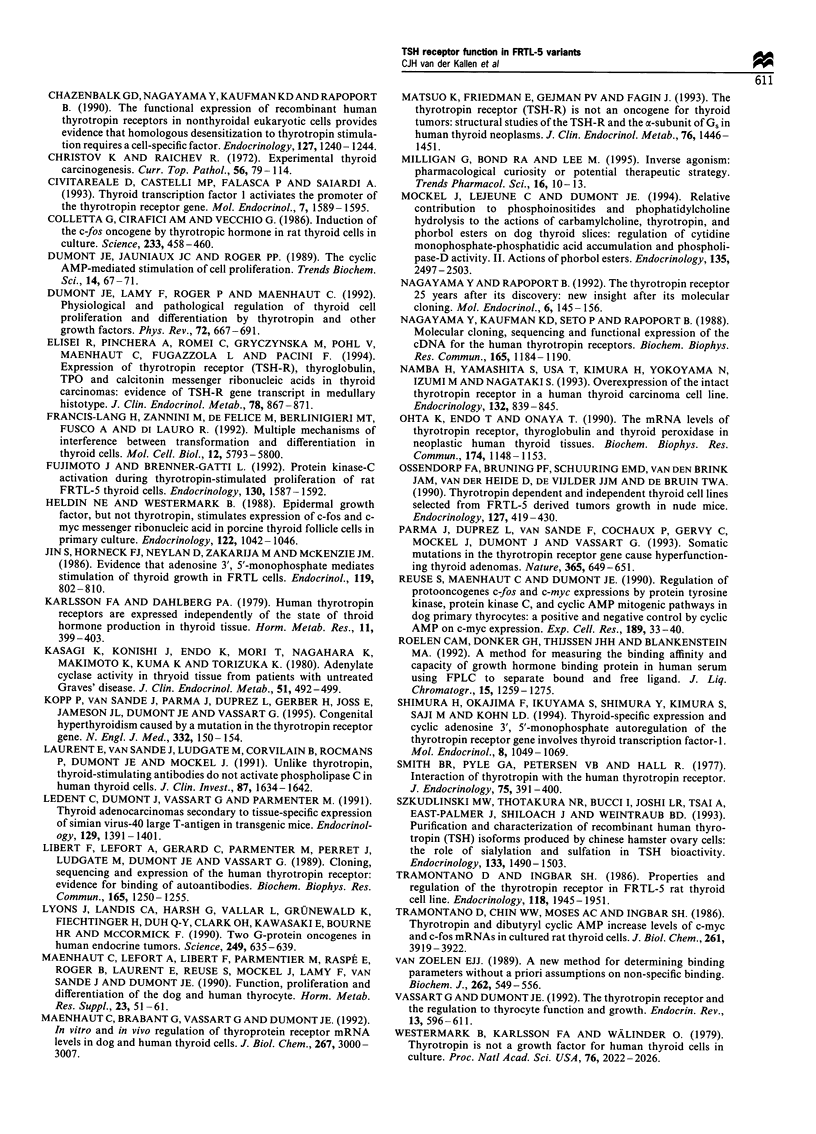

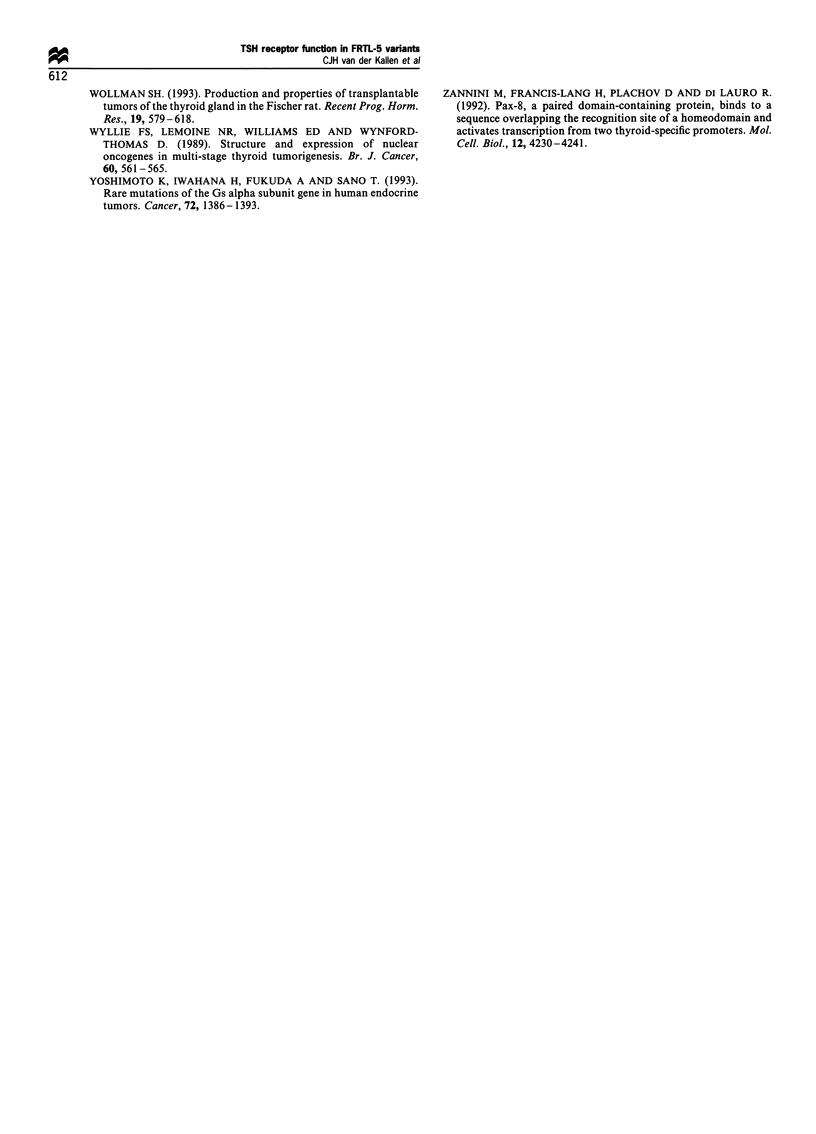


## References

[OCR_00695] Abe Y., Ichikawa Y., Muraki T., Ito K., Homma M. (1981). Thyrotropin (TSH) receptor and adenylate cyclase activity in human thyroid tumors: absence of high affinity receptor and loss of TSH responsiveness in undifferentiated thyroid carcinoma.. J Clin Endocrinol Metab.

[OCR_00703] Akamizu T., Ikuyama S., Saji M., Kosugi S., Kozak C., McBride O. W., Kohn L. D. (1990). Cloning, chromosomal assignment, and regulation of the rat thyrotropin receptor: expression of the gene is regulated by thyrotropin, agents that increase cAMP levels, and thyroid autoantibodies.. Proc Natl Acad Sci U S A.

[OCR_00706] Ambesi-Impiombato F. S., Parks L. A., Coon H. G. (1980). Culture of hormone-dependent functional epithelial cells from rat thyroids.. Proc Natl Acad Sci U S A.

[OCR_00723] BURTON K. (1956). A study of the conditions and mechanism of the diphenylamine reaction for the colorimetric estimation of deoxyribonucleic acid.. Biochem J.

[OCR_00714] Berlingieri M. T., Akamizu T., Fusco A., Grieco M., Colletta G., Cirafici A. M., Ikuyama S., Kohn L. D., Vecchio G. (1990). Thyrotropin receptor gene expression in oncogene-transfected rat thyroid cells: correlation between transformation, loss of thyrotropin-dependent growth, and loss of thyrotropin receptor gene expression.. Biochem Biophys Res Commun.

[OCR_00732] Chazenbalk G. D., Nagayama Y., Kaufman K. D., Rapoport B. (1990). The functional expression of recombinant human thyrotropin receptors in nonthyroidal eukaryotic cells provides evidence that homologous desensitization to thyrotropin stimulation requires a cell-specific factor.. Endocrinology.

[OCR_00736] Christov K., Raichev R. (1972). Experimental thyroid carcinogenesis.. Curr Top Pathol.

[OCR_00742] Civitareale D., Castelli M. P., Falasca P., Saiardi A. (1993). Thyroid transcription factor 1 activates the promoter of the thyrotropin receptor gene.. Mol Endocrinol.

[OCR_00745] Colletta G., Cirafici A. M., Vecchio G. (1986). Induction of the c-fos oncogene by thyrotropic hormone in rat thyroid cells in culture.. Science.

[OCR_00752] Dumont J. E., Jauniaux J. C., Roger P. P. (1989). The cyclic AMP-mediated stimulation of cell proliferation.. Trends Biochem Sci.

[OCR_00755] Dumont J. E., Lamy F., Roger P., Maenhaut C. (1992). Physiological and pathological regulation of thyroid cell proliferation and differentiation by thyrotropin and other factors.. Physiol Rev.

[OCR_00761] Elisei R., Pinchera A., Romei C., Gryczynska M., Pohl V., Maenhaut C., Fugazzola L., Pacini F. (1994). Expression of thyrotropin receptor (TSH-R), thyroglobulin, thyroperoxidase, and calcitonin messenger ribonucleic acids in thyroid carcinomas: evidence of TSH-R gene transcript in medullary histotype.. J Clin Endocrinol Metab.

[OCR_00772] Francis-Lang H., Zannini M., De Felice M., Berlingieri M. T., Fusco A., Di Lauro R. (1992). Multiple mechanisms of interference between transformation and differentiation in thyroid cells.. Mol Cell Biol.

[OCR_00777] Fujimoto J., Brenner-Gati L. (1992). Protein kinase-C activation during thyrotropin-stimulated proliferation of rat FRTL-5 thyroid cells.. Endocrinology.

[OCR_00782] Heldin N. E., Westermark B. (1988). Epidermal growth factor, but not thyrotropin, stimulates the expression of c-fos and c-myc messenger ribonucleic acid in porcine thyroid follicle cells in primary culture.. Endocrinology.

[OCR_00788] Jin S., Hornicek F. J., Neylan D., Zakarija M., McKenzie J. M. (1986). Evidence that adenosine 3',5'-monophosphate mediates stimulation of thyroid growth in FRTL5 cells.. Endocrinology.

[OCR_00794] Karlsson F. A., Dahlberg P. A. (1979). Human thyrotropin receptors are expressed independently of the state of thyroid hormone production in thyroid tissue.. Horm Metab Res.

[OCR_00800] Kasagi K., Konishi J., Endo K., Mori T., Nagahara K., Makimoto K., Kuma K., Torizuka K. (1980). Adenylate cyclase activity in thyroid tissue from patients with untreated Graves' disease.. J Clin Endocrinol Metab.

[OCR_00806] Kopp P., van Sande J., Parma J., Duprez L., Gerber H., Joss E., Jameson J. L., Dumont J. E., Vassart G. (1995). Brief report: congenital hyperthyroidism caused by a mutation in the thyrotropin-receptor gene.. N Engl J Med.

[OCR_00813] Laurent E., Van Sande J., Ludgate M., Corvilain B., Rocmans P., Dumont J. E., Mockel J. (1991). Unlike thyrotropin, thyroid-stimulating antibodies do not activate phospholipase C in human thyroid slices.. J Clin Invest.

[OCR_00818] Ledent C., Dumont J., Vassart G., Parmentier M. (1991). Thyroid adenocarcinomas secondary to tissue-specific expression of simian virus-40 large T-antigen in transgenic mice.. Endocrinology.

[OCR_00825] Libert F., Lefort A., Gerard C., Parmentier M., Perret J., Ludgate M., Dumont J. E., Vassart G. (1989). Cloning, sequencing and expression of the human thyrotropin (TSH) receptor: evidence for binding of autoantibodies.. Biochem Biophys Res Commun.

[OCR_00844] Maenhaut C., Brabant G., Vassart G., Dumont J. E. (1992). In vitro and in vivo regulation of thyrotropin receptor mRNA levels in dog and human thyroid cells.. J Biol Chem.

[OCR_00838] Maenhaut C., Lefort A., Libert F., Parmentier M., Raspé E., Roger P., Corvilain B., Laurent E., Reuse S., Mockel J. (1990). Function, proliferation and differentiation of the dog and human thyrocyte.. Horm Metab Res Suppl.

[OCR_00850] Matsuo K., Friedman E., Gejman P. V., Fagin J. A. (1993). The thyrotropin receptor (TSH-R) is not an oncogene for thyroid tumors: structural studies of the TSH-R and the alpha-subunit of Gs in human thyroid neoplasms.. J Clin Endocrinol Metab.

[OCR_00855] Milligan G., Bond R. A., Lee M. (1995). Inverse agonism: pharmacological curiosity or potential therapeutic strategy?. Trends Pharmacol Sci.

[OCR_00860] Mockel J., Lejeune C., Dumont J. E. (1994). Relative contribution of phosphoinositides and phosphatidylcholine hydrolysis to the actions of carbamylcholine, thyrotropin, and phorbol esters on dog thyroid slices: regulation of cytidine monophosphate-phosphatidic acid accumulation and phospholipase-D activity. II. Actions of phorbol esters.. Endocrinology.

[OCR_00876] Nagayama Y., Kaufman K. D., Seto P., Rapoport B. (1989). Molecular cloning, sequence and functional expression of the cDNA for the human thyrotropin receptor.. Biochem Biophys Res Commun.

[OCR_00871] Nagayama Y., Rapoport B. (1992). The thyrotropin receptor 25 years after its discovery: new insight after its molecular cloning.. Mol Endocrinol.

[OCR_00883] Namba H., Yamashita S., Usa T., Kimura H., Yokoyama N., Izumi M., Nagataki S. (1993). Overexpression of the intact thyrotropin receptor in a human thyroid carcinoma cell line.. Endocrinology.

[OCR_00886] Ohta K., Endo T., Onaya T. (1991). The mRNA levels of thyrotropin receptor, thyroglobulin and thyroid peroxidase in neoplastic human thyroid tissues.. Biochem Biophys Res Commun.

[OCR_00892] Ossendorp F. A., Bruning P. F., Schuuring E. M., Van Den Brink J. A., van der Heide D., De Vijlder J. J., De Bruin T. W. (1990). Thyrotropin dependent and independent thyroid cell lines selected from FRTL-5 derived tumors grown in nude mice.. Endocrinology.

[OCR_00899] Parma J., Duprez L., Van Sande J., Cochaux P., Gervy C., Mockel J., Dumont J., Vassart G. (1993). Somatic mutations in the thyrotropin receptor gene cause hyperfunctioning thyroid adenomas.. Nature.

[OCR_00905] Reuse S., Maenhaut C., Dumont J. E. (1990). Regulation of protooncogenes c-fos and c-myc expressions by protein tyrosine kinase, protein kinase C, and cyclic AMP mitogenic pathways in dog primary thyrocytes: a positive and negative control by cyclic AMP on c-myc expression.. Exp Cell Res.

[OCR_00922] Shimura H., Okajima F., Ikuyama S., Shimura Y., Kimura S., Saji M., Kohn L. D. (1994). Thyroid-specific expression and cyclic adenosine 3',5'-monophosphate autoregulation of the thyrotropin receptor gene involves thyroid transcription factor-1.. Mol Endocrinol.

[OCR_00928] Smith B. R., Pyle G. A., Petersen V. B., Hall R. (1977). Interaction of thyrotrophin with the human thyrotrophin receptor.. J Endocrinol.

[OCR_00934] Szkudlinski M. W., Thotakura N. R., Bucci I., Joshi L. R., Tsai A., East-Palmer J., Shiloach J., Weintraub B. D. (1993). Purification and characterization of recombinant human thyrotropin (TSH) isoforms produced by Chinese hamster ovary cells: the role of sialylation and sulfation in TSH bioactivity.. Endocrinology.

[OCR_00944] Tramontano D., Chin W. W., Moses A. C., Ingbar S. H. (1986). Thyrotropin and dibutyryl cyclic AMP increase levels of c-myc and c-fos mRNAs in cultured rat thyroid cells.. J Biol Chem.

[OCR_00939] Tramontano D., Ingbar S. H. (1986). Properties and regulation of the thyrotropin receptor in the FRTL5 rat thyroid cell line.. Endocrinology.

[OCR_00957] Vassart G., Dumont J. E. (1992). The thyrotropin receptor and the regulation of thyrocyte function and growth.. Endocr Rev.

[OCR_00970] WOLLMAN S. H. (1963). PRODUCTION AND PROPERTIES OF TRANSPLANTABLE TUMORS OF THE THYROID GLAND IN THE FISCHER RAT.. Recent Prog Horm Res.

[OCR_00962] Westermark B., Karlsson F. A., Wålinder O. (1979). Thyrotropin is not a growth factor for human thyroid cells in culture.. Proc Natl Acad Sci U S A.

[OCR_00977] Wyllie F. S., Lemoine N. R., Williams E. D., Wynford-Thomas D. (1989). Structure and expression of nuclear oncogenes in multi-stage thyroid tumorigenesis.. Br J Cancer.

[OCR_00981] Yoshimoto K., Iwahana H., Fukuda A., Sano T., Itakura M. (1993). Rare mutations of the Gs alpha subunit gene in human endocrine tumors. Mutation detection by polymerase chain reaction-primer-introduced restriction analysis.. Cancer.

[OCR_00986] Zannini M., Francis-Lang H., Plachov D., Di Lauro R. (1992). Pax-8, a paired domain-containing protein, binds to a sequence overlapping the recognition site of a homeodomain and activates transcription from two thyroid-specific promoters.. Mol Cell Biol.

[OCR_00952] van Zoelen E. J. (1989). Receptor-ligand interaction: a new method for determining binding parameters without a priori assumptions on non-specific binding.. Biochem J.

